# Kinetics of pulmonary immune cells, antibody responses and their correlations with the viral clearance of influenza A fatal infection in mice

**DOI:** 10.1186/1743-422X-11-57

**Published:** 2014-03-26

**Authors:** Jin Lv, Yanhong Hua, Dan Wang, Aofei Liu, Juan An, Aimin Li, Yanfeng Wang, Xiliang Wang, Na Jia, Qisheng Jiang

**Affiliations:** 1 The Second Artillery General Hospital, PLA, 16 Xinjiekouwai Street, Xicheng District Beijing 100088, China; 2 State Key Laboratory of Pathogen and Biosecurity, Beijing Institute of Microbiology and Epidemiology, 20 Dong-Da Street, Fengtai District Beijing 100071, P.R. China; 3 Department of Biochemistry, The University of Hong Kong, Pokfulam, Hong Kong, China

**Keywords:** Influenza A, Fatal infection, Macrophage, Neutrophil, Antibody

## Abstract

Fatal influenza A virus infection is a major threat to public health throughout the world. Lung macrophages and neutrophils have critical roles for both the pathogenesis and viral clearance of fatal viral infections. These are complicated by the interaction of innate immunity and adaptive immunity against viral infection. In this study, we investigated the overall kinetics of lung macrophages, neutrophils, CD4^+^T cells, CD8^+^T cells, CD38^+^ cells, and CD138^+^ cells, the levels of antibody and cytokine responses, both in the early and late phases of fatal infection with A/PR/8/34 (H1N1) virus in mice. The changes in lung viral load were also evaluated. We found that pulmonary macrophages and neutrophils both accumulated in the early and late phases of fatal infections and they positively correlated with the lung and serum antibody titers, and negatively correlated with the viral load locally. The secretion of IL-6 might relate to high numbers of macrophages and neutrophils in the early infection. The work implies that pulmonary macrophages, neutrophils and the antibody response all have an essential role in virus elimination of fatal influenza A viral infection. These findings may have implications for the development of prophylactic and therapeutic strategies in fatal influenza A viral infection. Further evaluation of the cooperation among macrophages, neutrophils and antibody responses in eliminating the virus with fatal infection is needed.

## Introduction

Influenza is an acute epidemic respiratory disease that results in a high rate of mortality in human beings, especially among the elderly and children. A large number of deaths due to influenza are reported every year around the world [1,2]. Clinically, influenza A virus is the most important virus among the three types of the influenza virus. Influenza A viruses belong to the family Orthomyxoviridae. On the basis of the antigenicity of their haemagglutinin (HA) and neuraminidase (NA) molecules, they are classified into 16 HA subtypes (H1–H16) and 9 NA subtypes (N1–N9). The point mutations and reassortment events of the viral genomes contribute to the emergence of new variants or strains with epidemic or pandemic potential [3]. Influenza A viruses have caused several pandemics during the last century, and continue to cause epidemics annually. The pandemic of 1918–1919 killed as many as 50 million people worldwide [4,5]. In 2009, a novel swine-origin influenza virus capable of rapid human transmission was reported. As of 4 April 2010, worldwide more than 213 countries and overseas territories or communities have reported laboratory confirmed cases of pandemic influenza H1N1 2009, including over 3200 deaths [6]. The recent report on the drug resistance to oseltamivir phosphate capsules [7] and H7N9 outbreak in China [8] have made the prevention and control of pandemic influenza more difficult. Therefore, understanding the mechanisms of increased pathogenicity of fatal influenza A viral infection is critical to optimize antiviral treatment strategies and control potential pandemics.

The uncontrolled and aberrant activation of the innate immune system has been implicated in the mice model of fatal influenza A viral infection [9-11]. A significantly rapid cell recruitment of macrophages and neutrophils into the lungs was assumed to have a role in the pathogenesis associated with H5N1 highly pathogenic avian influenza virus infection (HPAI) [12]. In addition, macrophages and neutrophils were associated with the increased secretion of some cytokine and chemokines [13], and increased levels of cytokines are suggested to mediate influenza A infection signs [14,15]. In addition, they may have a role in the severe symptoms of fatal HPAI H5N1 influenza virus infection [16-19]. However, inhibition of the cytokine response cannot protect against the lethal influenza A infection [20], and neutrophil or macrophage depletion in the early stage of infection has not had a significant effect on the outcome [13]. These findings have suggested complicated biological effects of macrophages and neutrophils in the fatal influenza A viral infection. In addition, innate immune cells such as macrophages and neutrophils, are the targets of influenza A viruses [12]. The direct infection of macrophages and neutrophils may seriously compromise the adaptive immune response.

The mouse model is very useful in the study of influenza virus pathogenesis, especially of the pneumonia by fatal infection, because the immune response and the correlations between these immune parameters in the lung can be monitored and evaluated directly. Influenza A/PR/8/34 H1N1 virus (PR8) is a mouse-adapted influenza strain, which induced the destruction of type II pneumocytes in alveoli in the mice [21,22]. In addition, several researchers used PR8 as the backbone virus to generate attenuated epidemic influenza vaccines [23-26]. Therefore, a detailed description on immune responses of PR8 fatal infection in the lung of mice could both contribute to the pathogenesis understanding and provide the useful data for comparison with reassortant influenza virus vaccine with PR8 backbone. In this study, we employed PR8 viruses to investigate the kinetics of innate and adaptive cellular immune responses in a mouse model. An overall picture of immune cell activities was obtained, both for the early and late phases of the fatal infection. The local viral load was also measured and its correlations with cellular responses and antibody levels were evaluated.

## Materials and methods

### Virus preparation

Influenza A/PR/8/34(H1N1) virus was kindly provided by Dr. Yuelong-Shu (Chinese Center for Disease Control and Prevention). Ten-day-old embryonated chicken eggs were infected with 0.1 ml of stock virus diluted to 1:1000 in PBS. After incubation for 48 hours at 35~36°C, the allantoic fluid was collected and clarified by centrifugation at 3500*g*_av_ for 20 min. Virus stocks were aliquoted and stored at −70°C until use. Fifty percent tissue culture infectious dose (TCID_50_) and 50% egg infectious dose (EID_50_) titers were determined by serial titration of viruses in Madin-Darby canine kidney (MDCK) cells and eggs, respectively. Titers were calculated by the method of Reed and Muench.

### Infection of mice

Female BALB/c mice were purchased from the Institute of Jingfeng Medical Laboratory Animal and were maintained under specific pathogen-free conditions. Five mice in each group were lightly anesthetized by ethylether inhalation and infected by intranasal inoculation (in 25 μl) of 5 × 10^5^, 5 × 10^4^, 5 × 10^3^, 5 × 10^2^, 5 × 10^1^ p.f.u. of viruses to determine the 50% lethal dose (LD_50_). Plaque assays were performed on MDCK cells to titration of the viruses. Plaque Forming Units (PFU) is a measure of the number of particles capable of forming plaques per unit volume, such as virus particles. It is a functional measurement rather than a measurement of the absolute quantity of particles: viral particles that are defective or which fail to infect their target cell will not produce a plaque and thus will not be counted. One plaque forming unit means a virus or group of viruses which cause a plaque. In the following experiment, 5 × 10^5^ p.f.u. viruses were used to lethal infection of 20 mice per group and eight groups were used. Mice not infected with influenza A/PR/8/34 virus were used as the control group. The animal experiments were approved by the Animal Subjects Research Review Board of the Beijing Institute of Microbiology and Epidemiology and were conducted according to the institution's guidelines for animal husbandry.

### Tissue preparation

Individual body weights from each group were recorded and monitored daily for disease signs and death for 14 d post infection. On days 2, 4, 6, 8, 10, 12 and 14 post infection, 15 mice at each time point were euthanized, and lungs were collected separately and homogenized in 2 mL sterile PBS. After this process, homogenates were frozen separately in sterile tubes at −80°C for later titration of antibody and cytokine detections. For the detection of lung immune cells, five to six mice at each time point post infection were sacrificed by cervical dislocation. The lungs were dissected and placed into cold DMEM. Lung cell preparations were made by passing tissue through a nylon screen. Red blood cells were removed by lysis buffer treatment (BD, biosciences). Cells were counted and resuspended at appropriate concentrations for each particular experiment.

### Flow cytometry and cytokine measurement

Single lung cell suspensions were stained with fluorochrome-labeled anti-CD3, anti-CD4, anti-CD8, anti-CD11b, anti-CD11c, anti-Ly-6G/C, anti-CD138 and anti-CD38 antibodies (BD Biosciences). Cells were labeled for 45 min at 4°C in staining buffer (PBS with 1%FBS, 0.02% NaN3), washed twice with PBS and fixed overnight at 4°C with 2% paraformaldehyde. Flow cytometry was performed on a FACS Aria flow cytometer (BD Biosciences). IL-6, IL-10, IL-17, IFN-γand IL-4 cytokine protein levels in the lungs were measured by specific enzyme-linked immunosorbent assay (ELISA; R&D Systems, Minneapolis, MN, USA).

### RT-PCR detection of the viral load

The viral load in the lung tissues was determined by the real-time reverse-transcriptase-polymerase-chain-reaction (RT-PCR). We extracted RNA from lung homogenates with Trizol (Invitrogen, USA) reagent. Then, reverse transcriptions targeting at a conserved region of influenza matrix (M) gene were performed, as previously described [27].

### Virus-specific Antibody assays

Influenza-specific serum and lung homogenate antibodies were measured by ELISA, using plates coated with 1 μg/ml per well of purified A/PR/8/34 (H1N1) influenza virus. Briefly, two-fold serial dilutions of sample (1:25 to1:3200 for IgA detection, 1:50 to 1:102400 for IgG detection) were incubated in the plates. Bound Ab was detected with HRP-conjugated goat anti-mouse Abs specific for IgG and IgA, and was developed with TMB (Sigma, USA). Absorbance was read at 450 nm on a Bio-RAD model 550 Microplate Reader. The virus-specific Ab titer was defined as the reciprocal of the highest sample dilution giving an absorbance value greater than twice that of the samples from the negative controls. The titers were gained in duplicate.

Haemagglutination inhibition (HI) assay was performed as previous described [28,29]. The HI titers were expressed as the reciprocal of the highest dilution that completely inhibited haemagglutination of erythrocyte.

### Statistical analysis

Statistical differences at each time point were determined by one-way ANOVA tests. A Spearman correlation analysis was performed to detect the correlation among viral load and frequencies of different cells, and antibody titers with SPSS19.0. Values of *p* <0.05 were considered significant.

## Results

**Figure 1:**
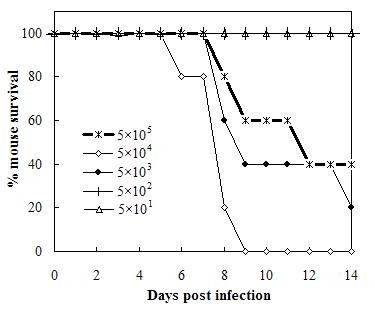
**Survival ratios of mice following intranasal infection with different doses of influenza A/PR/8/34 (H1N1) viruses.** Groups of five mice were infected intanasally with 5 × 10^5^ (*), 5 × 10^4^ (◇), 5 × 10^3^ (♦), 5 × 10^2^ (┼) or 5 × 10^1^ (△) p.f.u. A/PR/8/34 (H1N1) viruses and were observed consecutively for 14 d.

To investigate the lethal dose, we inoculated intranasally six groups of mice with 5 × 10^5^, 5 × 10^4^, 5 × 10^3^, 5 × 10^2^, 5 × 10^1^ p.f.u. of the viruses. In group of dose 5 × 10^4^ p.f.u., the first mouse death was observed at day 5, and the death rate increased to 100% at day 9 post infection (Figure 1). Compared with that group, the mice in group of dose 5 × 10^5^ p.f.u. began to die at day 7, and 40% mice survived until day 14. This dose induced severe pulmonary pathology (data not show) and was thus used in the following experiments to evaluate the immune responses in the lung of fatal influenza A/PR/8 virus infection.

### Comparing the kinetics of lung macrophage and neutrophils with other immune cells in lungs

**Figure 2:**
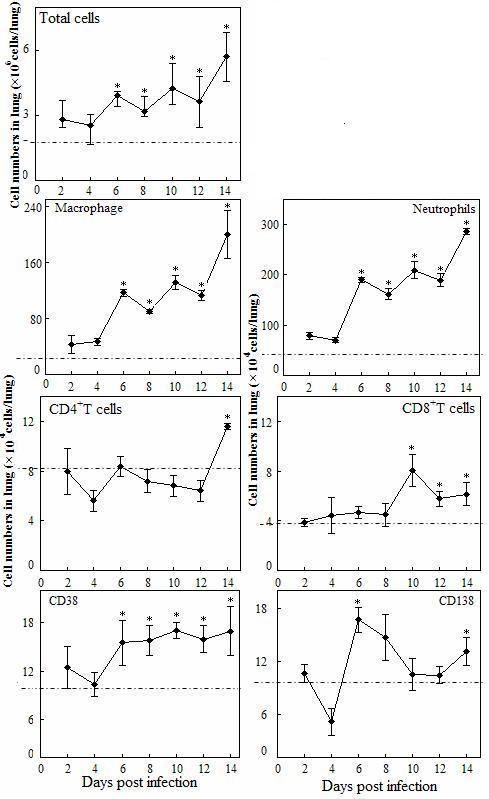
**Kinetics of pulmonary immune cells in mice with fatal dose (5 × 10**^**5 **^**p.f.u.) of A/PR/8/34 (H1N1) virus infection.** The frequencies of macrophages (CD11b^+^/CD11c^−^/Ly6G/c^−^) and neutrophils (CD11b^+^/CD11c^−^/Ly6G/c^+^) were determined by appropriate gating within the total lung leukocytes. For determination of the frequencies of CD4^+^ and CD8^+^ T cells, the CD3^+^ T cells were first determined by appropriate gating within the pulmonary lymphocytes, and then CD4^+^/CD8^−^T cells and CD4^−^/CD8^+^ T cells were detected by gating within the CD3^+^ T cells. In the detection of frequency of CD138^+^ cells and CD38^+^ cells, B220/CD45R was used to identify different B cell subtypes within the pulmonary lymphocytes, then CD138^+^ and CD38^+^ B cells were determined by appropriate gating within the determined B cell subtype, respectively. Mice without infection were used as the control group. Mean lung cell numbers were representative of at least 3 mice lungs per time point group, and error bars indicated the standard deviations. The dotted lines were the control level. *p < 0.05 compared with control group.

We first measured the frequency of lung macrophage, neutrophils, CD4^+^T cells, CD8^+^ T cells, CD38^+^cells and CD138^+^cells. The results shown in Figure 2 indicated that total pulmonary cells accumulated locally in A/PR/8/34 virus infection 6 days post infection, and kept a significant high level till 14 days post infection. Meanwhile, both the macrophages and neutrophils had a similar pattern (Figure 2). The increase of CD4^+^T cell was only observed in the late infection (14 days post infection) (Figure 2), while the increase of CD8 ^+^ T cells was observed from day 10 to 14 days post infection. However, the frequency of lung CD38^+^ increased from day 6 to 14 post infection, whereas CD138^+^ cells increased significantly at 6 days post infection, and then dropped to normal level later until day 14 post infection (Figure 2). In mice, CD38 is expressed on all naïve B cells but is down-regulated on isotype-switched B cells from germinal centers, foci of antibody-forming cells and mature plasma cells [29]. In contrast, CD138 is a cell surface heparan sulphate proteoglycan that is highly expressed by plasma cells.

### Comparing the kinetics of pulmonary macrophages and neutrophils with lung antibody responses in lung and serum of mice with fatal influenza A virus infection

**Figure 3:**
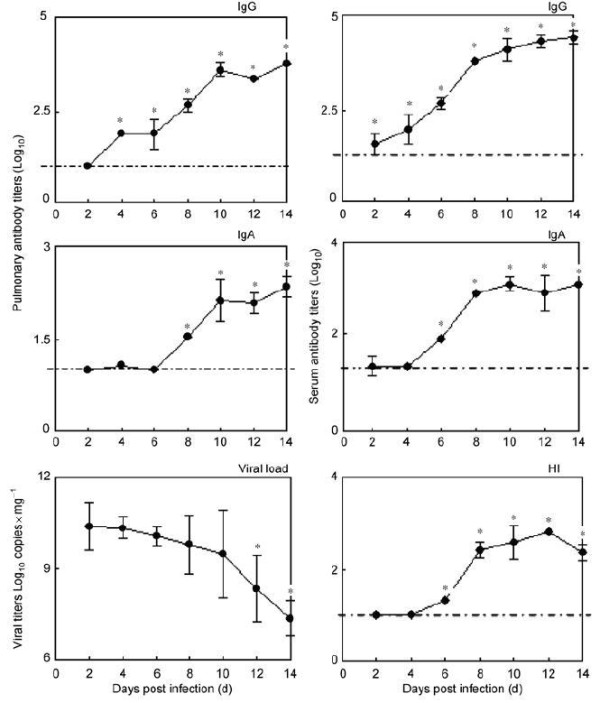
**The dynamics of pulmonary viral load and antibody response in lungs and serum in mice after fatal dose (5 × 10**^**5 **^**p.f.u.) of A/PR/8/34 (H1N1) virus infection per time-point.** HI, haemagglutination inhibition assay. Mice without infection were used as the control group. The dotted lines represented the results of control group. I bars represented standard deviations. *p < 0.05, compared with control group.

**Figure 4:**
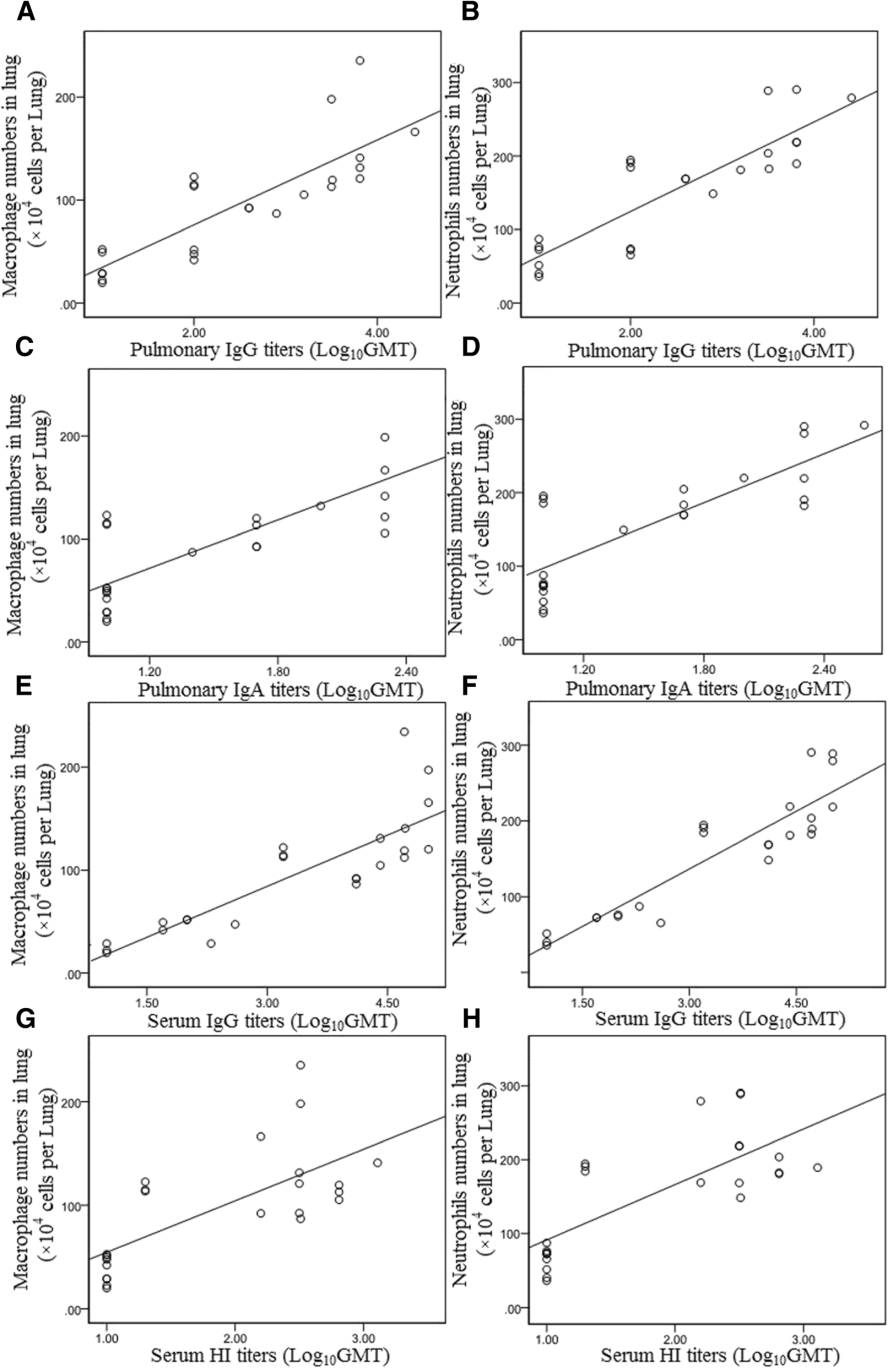
**Scatter plot of Table**1**. (A)** Scatter plot of macrophage numbers × 10^4^ per lung and pulmonary IgG titers of mice infected with A/PR/8/34 (H1N1) influenza viruses; **(B)** Scatter plot of neutrophils numbers × 10^4^ per lung and pulmonary IgG titers of mice infected with A/PR/8/34 (H1N1) influenza viruses; **(C)** Scatter plot of macrophage numbers × 10^4^ per lung and pulmonary IgA titers of mice infected with A/PR/8/34 (H1N1) influenza viruses; **(D)** Scatter plot of neutrophils numbers × 10^4^ per lung and pulmonary IgA titers of mice infected with A/PR/8/34 (H1N1) influenza viruses; **(E)** Scatter plot of macrophage numbers × 10^4^ per lung and serum IgG titers of mice infected with A/PR/8/34 (H1N1) influenza viruses; **(F)** Scatter plot of neutrophils numbers × 10^4^ per lung and serum IgG titers of mice infected with A/PR/8/34 (H1N1) influenza viruses; **(G)** Scatter plot of macrophage numbers × 10^4^ per lung and serum HI titers of mice infected with A/PR/8/34 (H1N1) influenza viruses; **(H)** Scatter plot of neutrophils numbers × 10^4^ per lung and serum HI titers of mice infected with A/PR/8/34 (H1N1) influenza viruses.

The dynamics of lung antibody levels were similar with the kinetics of lung macrophage and neutrophils frequencies (Figure 3). A correlation analysis showed that both macrophages and neutrophils closely correlated with IgG and IgA antibody responses in lungs (*R*^2^ = 0.826, *p* = 0.000 and *R*^2^ = 0.861, *p* = 0.000, *R*^2^ = 0.807, *p* = 0.000 and *R*^2^ = 0.810, *p* = 0.000, respectively), and serum IgG, and HI antibody responses (*R*^2^ = 0.844, *p* = 0.000 and *R*^2^ = 0.909, *p* = 0.000, *R*^2^ = 0.706, *p* = 0.000 and *R*^2^ = 0.750, *p* = 0.000, respectively), but no correlation with serum IgA antibody (*R*^2^ = 0.230, *p* = 0.280 and *R*^2^ = 0.323, *p* = 0.124, respectively) (Table 1). The scatter plots for the significant correlations see Figure 4.

**Table 1:** Correlations between antibody responses in lungs and serum and pulmonary macrophages and neutrophils in fatal mice infection

**Items**		**Macrophages**		**Neutrophils**	
		**R**^**2**^	***p***	**R**^**2**^	***p***
Lung	IgG	0.826	0.000**	0.861	0.000**
	IgA	0.807	0.000**	0.810	0.000**
Serum	IgG	0.844	0.000**	0.909	0.000**
	IgA	0.230	0.280	0.323	0.124
	HI	0.706	0.000**	0.750	0.000**

*p < 0.05, **p < 0.01 correlation between antibodies and macrophages or neutrophils.

### Correlations between viral load with pulmonary macrophages, neutrophils and antibody responses in lungs and serum

**Figure 5:**
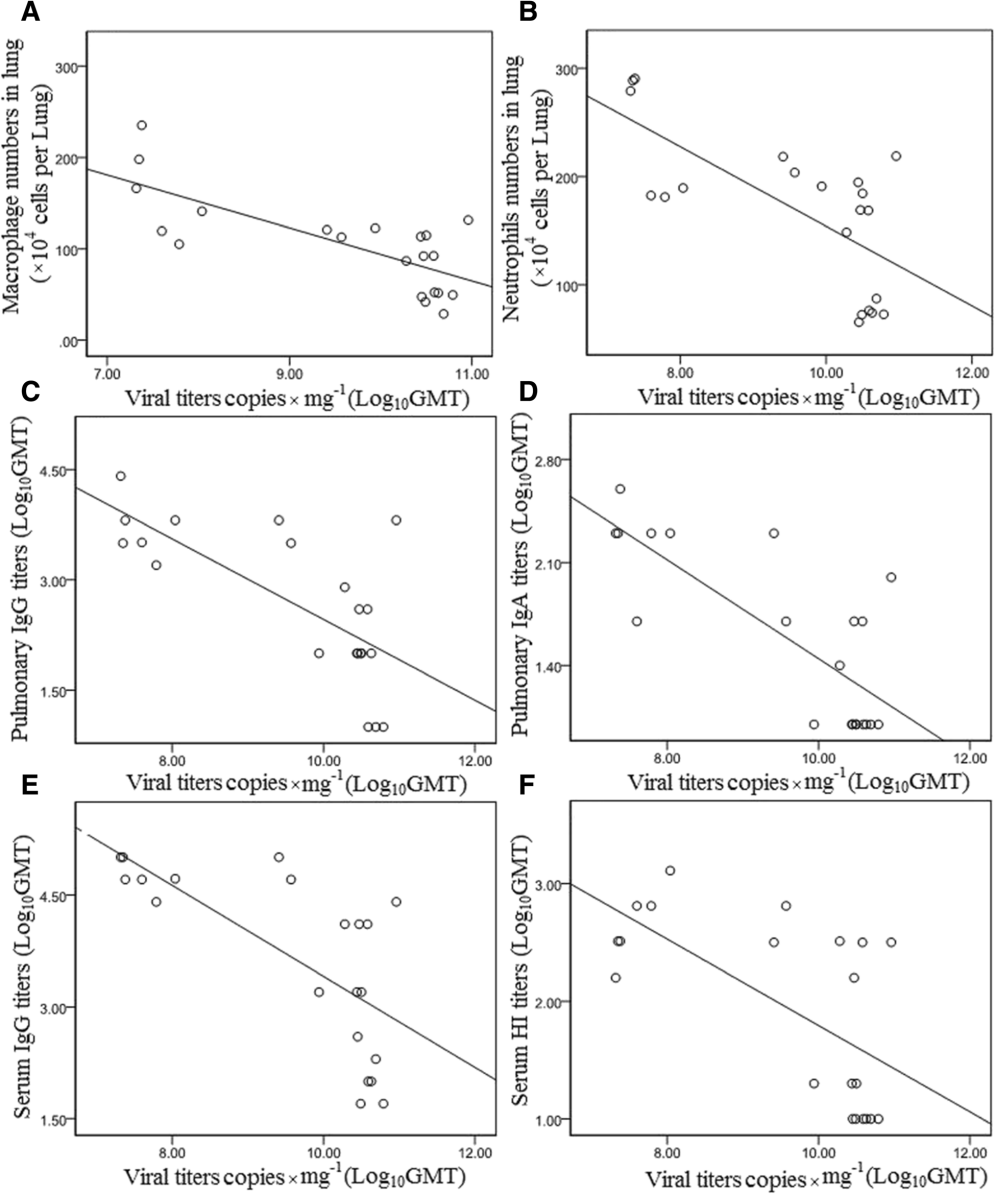
**Scatter plot of Table**2**. (A)** Scatter plot of macrophage numbers × 10^4^ per lung and viral copies × mg^−1^(Log_10_GMT) of mice infected with A/PR/8/34 (H1N1) influenza viruses; **(B)** Scatter plot of neutrophils numbers × 10^4^ per lung and viral copies × mg^−1^(Log_10_GMT) of mice infected with A/PR/8/34 (H1N1) influenza viruses; **(C)** Scatter plot of pulmonary IgG titers and viral copies × mg^−1^(Log_10_GMT) of mice infected with A/PR/8/34 (H1N1) influenza viruses; **(D)** Scatter plot of pulmonary IgA titers and viral copies × mg^−1^(Log_10_GMT) of mice infected with A/PR/8/34 (H1N1) influenza viruses; **(E)** Scatter plot of serum IgG titers and viral copies × mg^−1^(Log_10_GMT) of mice infected with A/PR/8/34 (H1N1) influenza viruses; **(F)** Scatter plot of serum HI titers and viral copies × mg^−1^(Log_10_GMT) of mice infected with A/PR/8/34 (H1N1) influenza viruses.

The influenza virus titer began to decrease in the late stage of infection. As indicated in Figure 3, at day 12 and day 14 post infection the virus significantly decreased. To measure whether the increased macrophage and neutrophil frequencies were correlated with the decrease of viral load, a correlation analysis was performed. Results showed that there was a significant negative correlation between macrophage or neutrophils and viral load in lungs (*R*^2^ = −0.751, *p* = 0.000 and *R*^2^ = −0.693 *p* = 0.000, respectively) (Table 2). In addition, the decreased viral loads significantly correlated with increased IgG antibody level both in the lungs and sera (*R*^2^ = −0.713, *p* = 0.000, and *R*^2^ = −0.695, *p* = 0.000, respectively), and with the increased lung IgA and sera HI antibody (*R*^2^ = −0.775, *p* = 0.000 and *R*^2^ = −0.637, *p* = 0.002, respectively) (Figure 3, Table 2). The scatter plots for the significant correlations see Figure 5. The decreased viral load showed no significant correlation with other immune cells (data not shown).

**Table 2:** Correlation analysis between viral load and pulmonary macrophages, neutrophils and antibody levels in serum and lungs in fatal mice infection

**Items**		**Viral load**	
		**R**^**2**^	***p***
Macrophages		−0.751	0.000**
Neutrophils		−0.693	0.000**
Lung	IgG	−0.713	0.000**
	IgA	−0.775	0.000**
Serum	IgG	−0.695	0.000**
	IgA	0.225	0.327
	HI	−0.637	0.002**

*p < 0.05, **p < 0.01 correlation between antibody and viral load and macrophages, neutrophils and antibodies in fatal mice infection.

### Kinetics of cytokines in the lung

**Figure 6:**
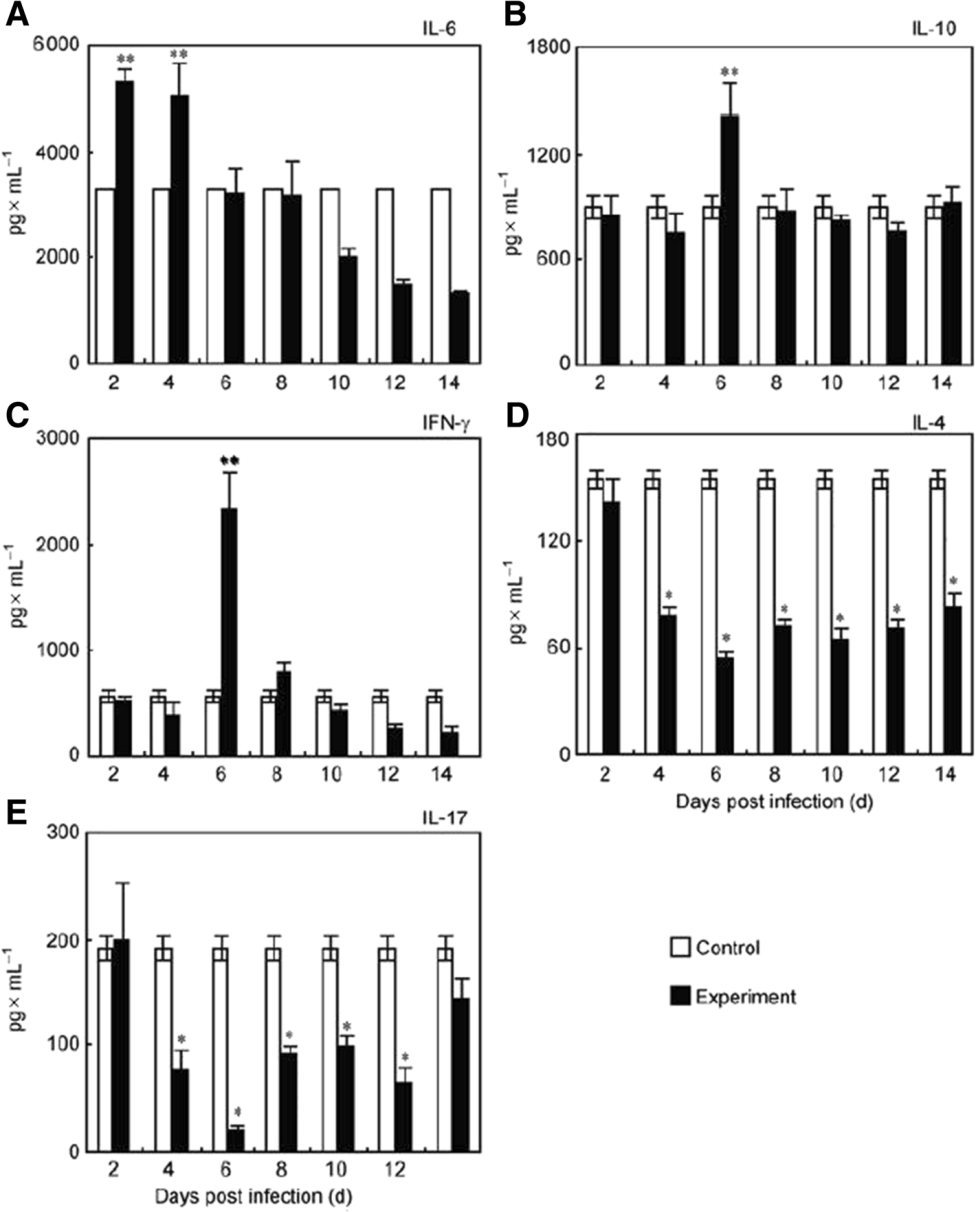
**Determination of cytokine levels in mice after fatal dose (5 × 10**^**5 **^**p.f.u.) of A/PR/8/34 (H1N1) virus infection.** IL-6 **(A)**, IL-10 **(B)**, IFN-γ **(C)**, IL-4 **(D)**, and IL-17 **(E)**. Mice without infection were used as the control group. The dotted lines represented the results of control group. Columns represented mean cytokine concentrations representative of 5~6 mice, and I bars indicated the standard deviations. *p < 0.05, compared with control group.

Three cytokines (IL-6, IFN-γ, IL-10) increased significantly in the early stage of infection, but they all decreased to control levels at the late stage. The earliest increased cytokine was IL-6, which increased at day 2 post-infection. Both IFN-γ and IL-10 significantly increased at day 6 post-infection, but all rapidly decreased to the control level later on. However, IL-17 and IL-4, showed a significant decreased level during the infection, especially after 2 days post infection (Figure 6).

### Correlations of cytokine responses with macrophages, neutrophils and viral load in lungs

**Figure 7:**
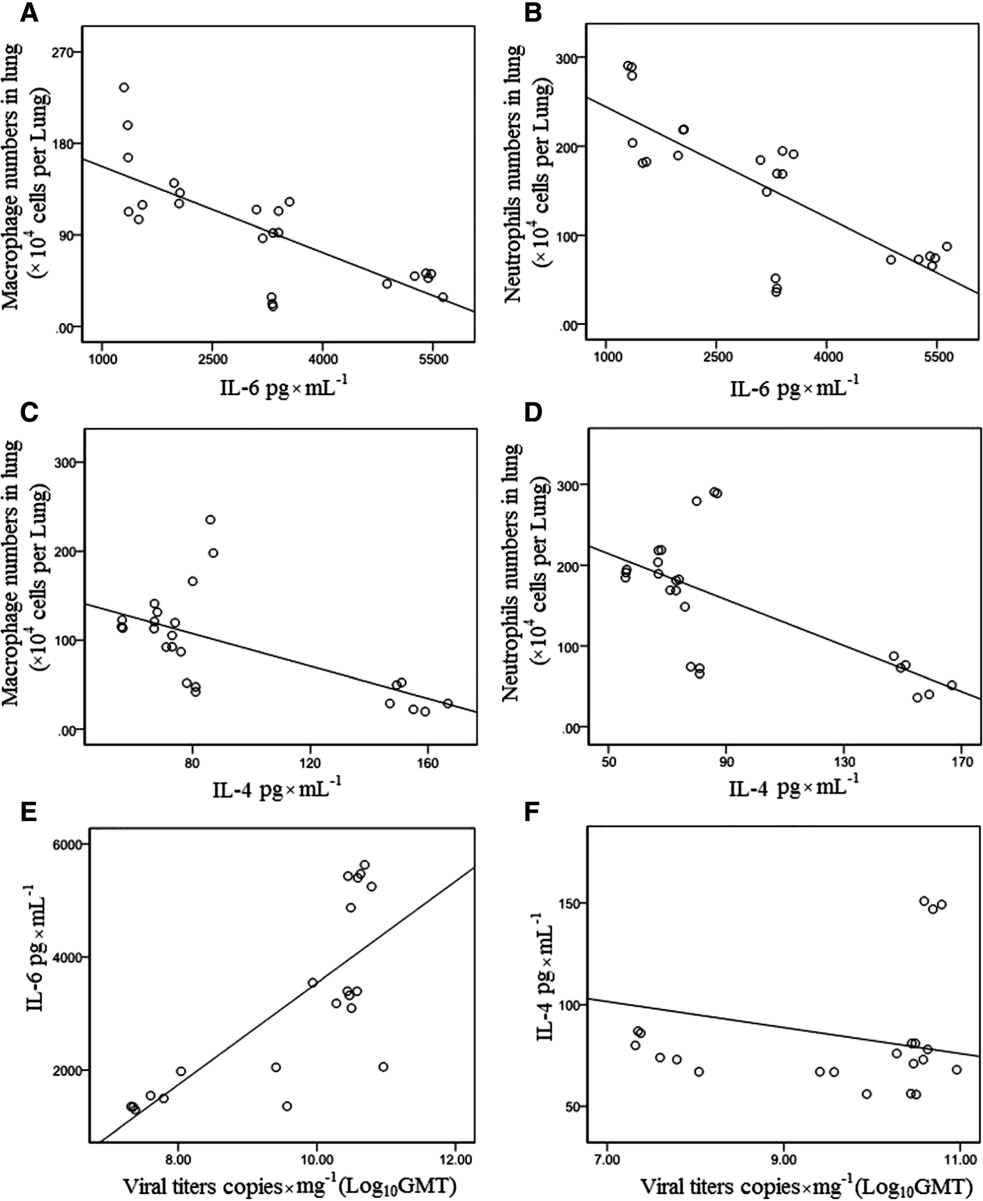
**Scatter plot of Table**3**. (A)** Scatter plot of macrophage numbers × 10^4^ per lung and pulmonary IL-6 pg × mL^−1^ of mice infected with A/PR/8/34 (H1N1) influenza viruses; **(B)** Scatter plot of neutrophils numbers × 10^4^ per lung and pulmonary IL-6 pg × mL^−1^ of mice infected with A/PR/8/34 (H1N1) influenza viruses; **(C)** Scatter plot of macrophage numbers × 10^4^ per lung and IL-4 pg × mL^−1^ of mice infected with A/PR/8/34 (H1N1) influenza viruses **(D)** Scatter plot of neutrophils numbers × 10^4^ per lung and pulmonary IL-4 pg × mL^−1^ of mice infected with A/PR/8/34 (H1N1) influenza viruses; **(E)** Scatter plot of pulmonary IL-6 pg × mL^−1^ and viral copies × mg^−1^(Log_10_GMT) of mice infected with A/PR/8/34 (H1N1) influenza viruses; **(F)** Scatter plot of pulmonary IL-4 pg × mL^−1^ and viral copies × mg^−1^(Log_10_GMT) of mice infected with A/PR/8/34 (H1N1) influenza viruses.

The dynamics of pulmonary cytokine response showed the different increase patterns. Statistical analysis showed that the decreased of IL-6 had a close relation with that of macrophages, neutrophils and the decrease of viral loads (*R*^2^ = −0.754, *p* = 0.000, *R*^2^ = −0.782, *p* = 0.000 and *R*^2^ = 0.759, *p* = 0.000, respectively) (Table 3). The significant negative correlations of IL-4 with macrophages and neutrophils and viral load were also observed (*R*^2^ = −0.612, *p* = 0.000, *R*^2^ = −0.673, *p* = 0.000 and *R*^2^ = −0.592, *p* = 0.000, respectively), while no close correlation was observed between other cytokines and cells (*P* > 0.05) (Table 3). The scatter plots for the significant correlations see Figure 7.

**Table 3:** Correlations among the cytokine responses, macrophages, neutrophils and viral load in lungs in fatal mice infection

**Cytokines**	**Macrophages**		**Neutrophils**		**Viral load**	
	**R**^**2**^	***p***	**R**^**2**^	***p***	**R**^**2**^	***p***
IL-6	−0.754	0.000**	−0.782	0.000**	0.759	0.000**
IL-4	−0.612	0.000**	−0.673	0.000**	−0.592	0.002**
IFN-γ	0.060	0.780	0.119	0.581	0.310	0.171
IL-10	0.143	0.504	0.196	0.360	0.150	0.516
IL-17	−0.235	0.268	−0.250	0.238	−0.048	0.837

*p < 0.05, **p < 0.01 correlation between cytokines and macrophages, neutrophils and viral load.

## Discussion

Macrophages and neutrophils have a complicated role in protecting against high-dose lethal infection of influenza A virus. In this study, the mice were fatally infected with A/PR/8/34 (H1N1) influenza virus. We monitored the kinetics of the macrophages, neutrophils, CD4^+^T cell, CD8^+^ T cell, CD138^+^cell and CD38^+^cell in the lungs during the viral infection. We found that macrophages and neutrophils accumulated in the lungs, both in the early and late phases of the virus infection. The decreased viral load significantly correlated with the increased macrophages and neutrophils, and antibody levels both in the lung and serum. The present study suggests that macrophages and neutrophils and the antibody responses both have an important role in eliminating of influenza virus locally.

As to both induce the fatal immunopathology in the lung and ensure the enough mice to survive for further experiment, we at first established an appropriate dose of virus. We found 5 × 10^5^ p.f.u. viruses had a decreased lethality in mice than the dose of 5 × 10^4^ and 5 × 10^3^ p.f.u. The reason might be that 5 × 10^4^ p.f.u. could replicate more efficiently than 5 × 10^5^p.f.u.viruses in the upper respiratory tract, thus came higher lethality. We understood it was not common for other influenza viruses, and did not know whether it was correlated with the characteristic of PR8 as a mouse-adapted virus strain, although there was no published article reported the similar experiments.

Observations from de Jong *et al*. [30] supported the presence of an inflammatory response role in the pathogenesis of human H5N1 disease. In addition, post-mortem studies in H5N1-infected individuals have not shown predominance of lymphocytes, but rather of macrophages, in pulmonary in-filtrates [31]. Although CD4^+^T cell and CD8^+^T cells also have a protective effect during the pathogen infection, especially during high pathogenic avian influenza viruses infection, the protective was also seen in the absence of all T and B cells as well as in the depletion of neutrophils or NK cells [32]. Whereas, depletion of innate lymphoid cells resulted in loss of airway epithelial integrity, diminished lung function and impaired airway remodeling [33]. Furthermore, study by Tate MD [34] showed that neutrophil depletion early after infection with influenza virus did not alter influenza virus-derived antigen presentation or naïve CD8^+^T-cell expansion in the secondary lymphoid organs and of trafficking of virus-specific CD8^+^T cells into the infected pulmonary airways. Instead early neutrophils reduced both the overall magnitude of influenza virus-specific CD8^+^T cells, together with impaired cytokine production and cytotoxic effector function. In contrast to that, the depletion of macrophages lead to the death of all of the mice even those challenged with a sublethal dose of virus [32], therefore it was impossible to evaluate the protective effect with removal of macrophages.

The accumulation of macrophages and neutrophils in the early phase also have been observed in mice [12] and chickens [35] fatally infected with influenza viruses. In this study, we observed a significant increased frequency of macrophages in the lung at day 4 post infection. We also evaluated the kinetics of macrophages and neutrophils in the late phase infection. When the viral load in the lung significantly decreased at day 10 post infection, the macrophage and neutrophil frequencies were maintained at high levels. Previous research also found that in the late recovery phase, macrophage and neutrophil inhibition led to a marked delay in the elimination of the virus. All these observations suggest that macrophages and neutrophils may contribute to late-phase clearance of influenza viruses [36]. Previous evidence has suggested that influenza A virus infected cells are subjected to apoptosis-dependent phagocytosis and degrade together with the invading virus within phagocytes. It has been speculated that macrophages and neutrophils accumulate in lung tissues and maximize the efficiency of the phagocytic elimination of infected cells [37].

IL-6 secretion significantly increased early at day 2 post-infection and quickly decreased to the level of the control, suggesting a correlation with the accumulation of macrophage. Suzuki [35] also reported that IL-6 mRNA was quickly induced at 24 h post-infection, but 8 h later, mRNA levels became dramatically lower. IL-6 concentrations were significantly correlated to the symptoms and signs of influenza A infection in humans [14,15]. Elevated IL-6 values have been detected in HPAI H5N1 influenza virus infected human cells and mice [16]–[19,38]. As a multifunctional cytokine expressed by both lymphoid and non-lymphoid cells [39], IL-6 has a central role in elucidating an innate immune response and directing the transition from innate to adaptive immunity [40]. However, IL-6 cytokine inhibition does not directly protect against death from lethal H5N1 influenza virus infection [20].

Our study evaluated kinetic responses and correlation of several types of immune cells, cytokines and antibodies in a mouse model, but did not reveal how they were orchestrated in the virus clearance. Further evaluation of the cooperation between macrophages, neutrophils and antibody responses in eliminating the virus with fatal infection is needed.

## Competing interests

The authors declare that they have no competing interests.

## Author’s contributions

JL, YHH, XLW, QSJ and NJ conceived and designed the study. Experiments were performed by JL, YHH, DW, and AFL. Data were analysed by JL, AJ, AML, YFW, and NJ. JL and NJ participated in its design and coordination and helped to draft the manuscript. All authors read and approved the final manuscript.
